# An Analysis of Fluid Intake Assessment Approaches for Fluid Intake Monitoring System

**DOI:** 10.3390/bios14010014

**Published:** 2023-12-25

**Authors:** Chia-Yeh Hsieh, Hsiang-Yun Huang, Chia-Tai Chan, Li-Tzu Chiu

**Affiliations:** 1 Bachelor’s Program in Medical Informatics and Innovative Applications, Fu Jen Catholic University, New Taipei City 24205, Taiwan; 2 Department of Biomedical Engineering, National Yang Ming Chiao Tung University, Taipei City 11221, Taiwan; shoyhuang.y@nycu.edu.tw (H.-Y.H.); ctchan@nycu.edu.tw (C.-T.C.); joy40633@gmail.com (L.-T.C.)

**Keywords:** fluid intake assessment, wrist-worn sensor, smart-container sensor, gesture recognition, volume estimation

## Abstract

Monitoring fluid intake is essential to help people manage their individual fluid intake behaviors and achieve adequate hydration. Previous studies of fluid intake assessment approaches based on inertial sensors can be categorized into wrist-worn-based and smart-container-based approaches. This study aims to analyze wrist-worn-based and smart-container-based fluid intake assessment approaches using inertial sensors. The comparison of these two approaches should be analyzed according to gesture recognition and volume estimation. In addition, the influence of the fill level and sip size information on the performance is explored in this study. The accuracy of gesture recognition with postprocessing is 92.89% and 91.8% for the wrist-worn-based approach and smart-container-based approach, respectively. For volume estimation, sip-size-dependent models can achieve better performance than general SVR models for both wrist-worn-based and smart-container-based approaches. The improvement of MAPE, MAD, and RMSE can reach over 50% except MAPE for small sip sizes. The results demonstrate that the sip size information and recognition performance are important for fluid intake assessment approaches.

## 1. Introduction

Water is vital for all living cells in the human body. To maintain the balance of body fluids, enough water and fluid intake are essential to human beings. Fluid intake is crucial for kidney function, regulating body temperature, transporting nutrients, and supporting metabolism [1,2,3]. The daily recommended water intake for one person varies widely due to several factors, such as age, gender, physical activity level, climate, and dietary contents [4]. According to the European Food Safety Authority [5], adequate water intake is at least 2 L per day for women and 2.5 L per day for men, while U.S. National Academy of Medicine guidelines recommend a higher water intake volume (at least 2.7 L/day for women and 3.7 L/day for men) [6]. Inadequate water or fluid intake may induce dehydration (excessive fluid losses) or overhydration (excessive fluid intake). Dehydration and overhydration can result in various health issues, including headaches, fatigue, dizziness, edema, and hyponatremia [7,8]. Therefore, assessing and monitoring water or fluid intake are essential to help individuals understand and manage their personal fluid consumption for achieving adequate hydration [9].

Cameras, inertial sensors, acoustic sensors, and pressure sensors are commonly used in fluid intake assessment and monitoring approaches [10]. Based on the characteristics of these devices, fluid intake assessment and monitoring approaches can be categorized into three main groups: environment-based, wrist-worn-based, and smart-container-based approaches. Environment-based approaches usually use cameras or pressure sensors fixed in environments to collect data and identify drinking activities [11,12,13,14]. In wrist-worn-based approaches, individuals wear sensors on their right or left wrists to collect activity data. Detection algorithms are then applied to recognize drinking activities from the collected data [2,15,16]. In smart-container-based approaches, sensors are either attached to or embedded within a container to record data. These sensors come in various types, including inertial sensors [17,18,19], pressure sensors [20], capacitive sensors [19], and conductive sensors [21]. However, each approach has its own advantages and disadvantages. The main disadvantage of environment-based approaches is confined environments, which make the environment-based approaches unfeasible in un-sensing or uncontrol environments. In contrast, wrist-worn-based and smart-container-based approaches have no environmental restrictions. They can acquire the related data anytime and anywhere. Among the studies focusing on wrist-worn-based and smart-container-based approaches, inertial sensors are widely adopted as signal acquisition devices due to their advantages, such as small size, lightweight design, and unobtrusiveness. Most previous studies [2,10,15,17,18,19,22] have proposed these approaches and demonstrated their effectiveness in assessing fluid intake using inertial sensors.

Although both wrist-worn-based and smart-container-based approaches utilize inertial sensors, the captured signals differ from each other during drinking activities. Inertial sensors in wrist-worn-based approaches are worn on the wrist to sense wrist motion. In contrast, sensors in smart-container-based approaches are typically attached to the bottom of containers to detect container movement. As the wrist-worn sensor presents more complex signals, individual differences may cause larger effects on wrist-worn-based approaches than smart-container-based approaches. It is important to compare and analyze the performance of fluid intake recognition for these two approaches. However, to our knowledge, previous studies only used a single wrist-worn sensor or a single smart-container sensor to collect data and monitor fluid intake. Therefore, there is a need for a comprehensive analysis of these two approaches.

This study aims to analyze wrist-worn-based and smart-container-based fluid intake assessment approaches using inertial sensors in the same experimental scenario. We will compare the performance of gesture recognition and volume estimation in both wrist-worn-based and smart-container-based fluid intake assessment approaches. Additionally, we will explore how fill level and sip size information impacts performance. In each approach, we have developed a hierarchical algorithm that not only recognizes drinking gestures but also estimates fluid intake volumes. A typical drinking activity involves a series of gestures, including grasping the container, lifting it, taking a sip, placing it back down, and releasing it. Gesture recognition is employed to identify each specific gesture within a drinking activity, providing detailed information highly correlated with intake volumes. Volume estimation relies on factors like inclination and fill level to train regression models. Additionally, we have implemented models tailored to different sip sizes to enhance the accuracy of estimation.

The main contributions of this study are listed as follows:

- Experimental scenario: This study designs the experimental scenario for unbiased conditions and the same foothold. In the experiment, the motion data are collected from sensors on the wrist and in the container simultaneously during the whole drinking activity. This study analyzes and compares performances of two approaches through synchronous data collection for providing reliable referenced information.
- Consideration of fill levels and sip sizes: To enhance the reliability and robustness of fluid intake assessment, this study factors in variables such as fill levels in containers and sip sizes. These factors can influence the tilt of the container and the rotation of the wrist, but they have often been overlooked in previous research. This study conducts an in-depth analysis of how fill levels and sip sizes affect the estimation performance of both approaches.

The rest of this study is organized as follows: we briefly introduce related works of wrist-worn-based approaches and smart-container-based approaches in Section 2. The experimental protocol is described in Section 3. The proposed fluid intake assessment approaches for the wrist-worn-based approach and smart-container-based approach are illustrated in Section 4. In Section 5, the experimental results are demonstrated, including the performance of gesture recognition and volume estimation. The effect and potentiality of the proposed approach are discussed in Section 6. Finally, the conclusion is presented in Section 7.

## 2. Related Work

Many studies have proposed approaches to monitor fluid intake using various sensors. Considering the mobility of sensors, wrist-worn-based and smart-container-based approaches are common for fluid intake assessment. The important topics of these studies are reviewed in this section, as shown in Table 1.

In the wrist-worn-based assessment approach, data are collected from the wrist-worn inertial sensor. However, these data includes complex hand activities, such as drinking, eating, typing, combing, and calling, which may affect the performance of detecting drinking activities in the wrist-worn-based assessment approach. To distinguish drinking activities from complex hand activities and improve the estimation’s reliability, the wrist-worn-based assessment approach focuses on recognizing drinking activities. This recognition commonly employs statistical models [2,23], machine learning techniques [16,22,23] and deep learning techniques [15] to classify drinking activities and other hand activities. These studies showed that using the random forest learning technique to recognize drinking activity can obtain sensitivity in excess of 90% [16,23]. To enhance intake volume estimation, it is crucial to capture detailed information about drinking gestures, particularly the sip gesture. Therefore, we propose a drinking gesture recognition system that identifies key drinking gestures, including grasping the container, lifting it, sipping, placing it down, and releasing it. According to the previous study [22], using the support vector machine (SVM) technique can achieve the best sensitivity for distinguishing the sip gesture. In addition, volume estimation using machine learning regression models can achieve good performance. Commercial wrist-worn devices, such as smartwatches and smart bands, are common examples of wearable technology. These devices often feature inertial sensors and optical sensors to measure motion data and heart rates. In addition to commercial products, researchers and industries can utilize research-grade and industrial-grade inertial sensors, such as Opal^®^ sensors (manufactured by APDM Wearable Technology of ERT, Portland, OR, USA) and Mtw Awinda sensors (produced by Xsens Technologies, Enschede, The Netherlands), to record motion data for drinking activity recognition [22,26]. 

In the smart-container-based assessment approach, recording data only encompass drinking and drinking-related activities specific to a particular container. The complexity of activities in the smart-container-based assessment approach is lower compared to the wrist-worn-based assessment approach. However, the smart-container-based approach has restrictions, e.g., only using the smart container to drink. To estimate the intake amount, the smart-container-based assessment approach employs regression models, including linear regression and support vector machine regression (SVR) [18,19,24]. Studies have demonstrated that utilizing the SVR technique with Gaussian kernel functions can yield accurate estimation results [18,24]. Additionally, techniques such as micro-event partitioning and fill level classification are employed to enhance performance [18,24]. Micro-event partitioning isolates the lifting, sipping, and placing phases of a drinking event, while fill level classification provides initial fill level data, which can influence the container’s tilt angle during drinking. Commercial smart bottles, such as H2OPal (developed and produced by Out of Galaxy, Inc., Wilmington, DE, USA), HidrateSpark Steel, HidrateSpark 3 (developed and produced by Hidrate Inc., Minneapolis, MN, USA), and Thermos Smart Lid (developed and produced by Thermos LLC, Schaumburg, IL, USA), are available [19]. H2Opal and HidrateSpark Steel use pressure sensors in their bases to measure the amount, while HidrateSpark 3 and Thermos Smart Lid use capacitive sensors for volume evaluation. Research-grade smart containers are created using 3D-printed cups with embedded inertial sensors [25].

## 3. Experimental Protocol

**Figure 1:**
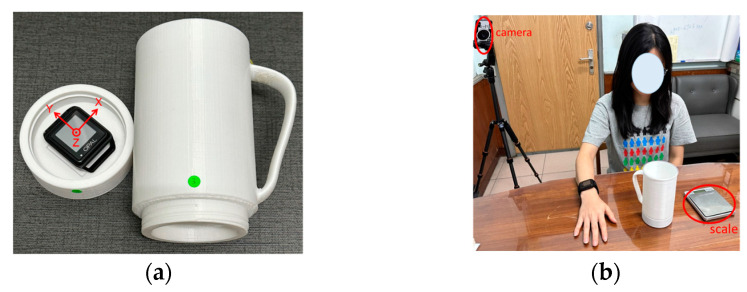
(**a**) The 3D-printed cup and the orientation of the OPAL sensor. (**b**) The experimental environment setting includes the wrist-worn sensor, a 3D-printed cup, a scale, and a synchronized camera.

Two Opal sensors, which were published by APDM, Portland, USA, are utilized to acquire the data. One is worn on the right wrist and the other is attached to the bottom of a 3D-printed cup, as shown in Figure 1. The tri-axial acceleration (range ±16 G) and angular velocity (range ±2000 degree/s) are collected with a sampling rate of 128 Hz. The orientation of the sensor is shown in Figure 1a. A camera embedded in the smartphone and synchronized with Opal sensors is applied to record video during the whole experiment to provide the ground truth of time labels for gesture recognition. A scale is used to measure the weight of each sip for obtaining true sip amounts. The experimental environment setting is shown in Figure 1b.

**Figure 2:**
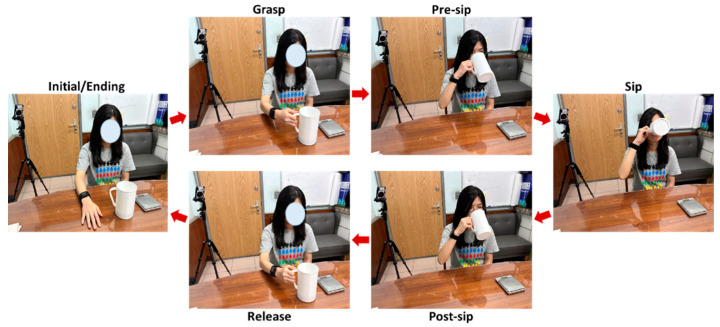
The performed gesture sequence including grasping the container (*grasp*), lifting the container (*pre-sip*), sipping (*sip*), putting down the container (*post-sip*), and releasing the container (*release*).

Twelve participants are recruited for this study, including six males and six females (age: 22.42 ± 0.67 years; height: 167.17 ± 7.53 cm; weight: 69.08 ± 19.14 kg). Each participant is asked to sit on the chair and take the specific cup to drink water with the right hand. The full capacity of the specific cup is 500 g. To consider real-world drinking situations, fill levels and sip sizes are investigated in the experiment. Seven fill levels (100 g, 150 g, 200 g, 250 g, 300 g, 350 g, and 400 g of water) and three sip sizes (small, medium, and large sip sizes) are applied. The performed drinking event contains a sequence of gestures, as shown in Figure 2. Initially, the participant places their right hand on the table. Then, the participant moves their hand to take the container (*grasp*). After holding the container, the participant lifts the container towards their mouth (*pre-sip*). While the container touches the mouth, the participant takes a sip with the specified sip size (*sip*). After sipping, the participant puts down the container on the table (*post-sip*). Finally, the participant releases the container (*release*). Each trial of the same fill level and sip size is repeated four times. For every trial, the true sip amount is recorded by a scale. The total number of drinking events per participant is 84 (7 fill levels × 3 sip sizes × 4 times).

## 4. Methodology

**Figure 3:**
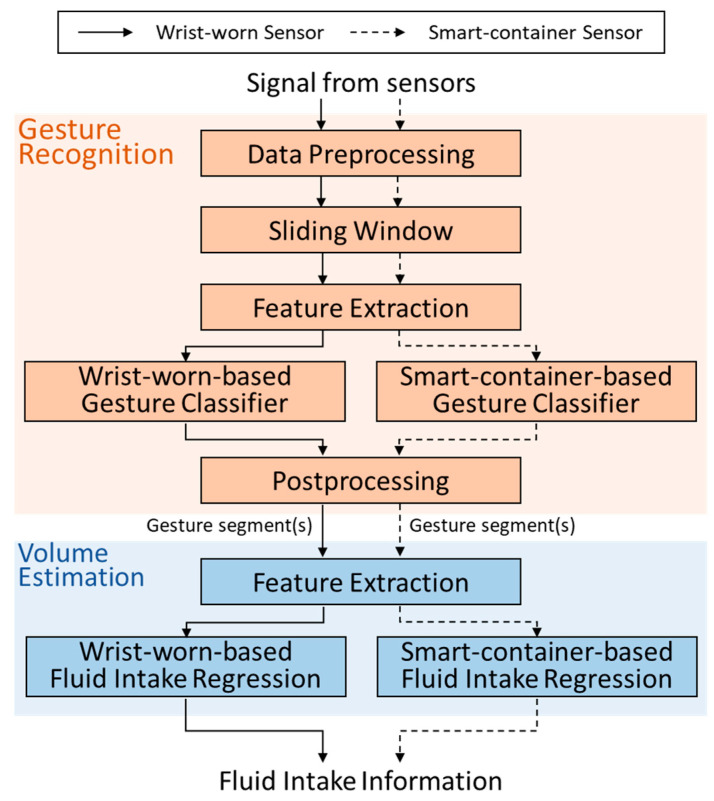
The flow diagram of wrist-worn-based and smart-container-based fluid intake assessment approaches. The hierarchical algorithm consists of gesture recognition and volume estimation.

The flow diagram of wrist-worn-based and smart-container-based fluid intake assessment approaches is shown in Figure 3. The hierarchical algorithm consists of gesture recognition and volume estimation. Firstly, the signals are collected from the wrist-worn and smart-container sensors synchronously in the experiment. Then, gesture recognition uses the machine-learning-based gesture classifier to identify the gesture segments from the drinking activity. Finally, volume estimation applies the regression model to estimate the amount of water in a drink activity by the identified gesture segments.

### 4.1. Gesture Recognition

In gesture recognition, the whole signal is separated into gesture segments to provide critical gesture information for volume estimation. Gesture recognition algorithm can be divided into five steps, including data preprocessing, sliding window, feature extraction, gesture classifier and postprocessing. Firstly, data preprocessing extracts drinking activities manually from the whole collected data. A total of 1008 drinking activities are obtained. Secondly, the sliding window technique segments continuous data into several fragments. The window size and overlap percentage in the sliding window technique are important parameters, which may affect the feature extraction from sequences of data and the performance of classifiers [27,28,29,30]. However, there is no precise guideline for selecting the optimal window size. A larger window might encompass multiple activities, while a smaller one could separate a single activity into pieces. Therefore, finding the suitable window size is crucial. Overlapping windows can prevent the miss of movement data and enable finer classification in smaller time intervals, but they demand more computational resources compared to non-overlapping windows. In this study, window sizes of 16, 24, 32, 40, 48, and 56 data samples with overlap percentages of 25, 50, 75, and 87.5% are investigated to achieve the best performance for gesture recognition. The third step is feature extraction. Eight statistical features are applied, including mean, standard deviation, variance, maximum, minimum, range, skewness, and kurtosis. Each type of feature is extracted from tri-axial acceleration, angular velocity, angular acceleration, and inclination. A total of 96 features are extracted in this study, as shown in Table 2.

The fourth step is gesture classifier, which aims to recognize detailed gestures from a drinking activity. A drinking activity is defined as a combination of gestures, including grasping the container (*grasp*), lifting the container (*pre-sip*), sipping (*sip*), putting down the container (*post-sip*), and releasing the container (*release*). Gesture classifier utilizes a support vector machine (SVM) model with radial basis function (RBF) kernel to recognize these gestures. In the wrist-worn-based gestures classifier, these five gesture segments can be recognized. However, only singles of *pre-sip*, *sip*, and *post-sip* can be captured in the smart-container-based gestures classifier. When the sensor is attached to the bottom of the container, only the signals that related to moving the container can be collected. Therefore, only signals of pre-sip, sip, and post-sip can be captured in the smart-container-based gesture classifier.

**Figure 4:**
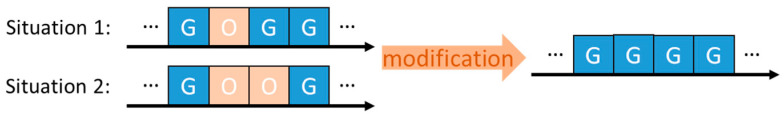
An example of postprocessing. If the predicted results of one fragment (situation 1) or two consecutive fragments (situation 2) differ from that of the preceding and subsequent fragments, and the predicted results of the adjacent fragments remain the same, the fragment is considered misclassified and adjusted to match the predicted results of the preceding fragment. G represents target gestures and O represents other gestures.

Finally, postprocessing modifies the misclassified fragments from the gesture classifier. The sliding window technique segments the stream data into multiple fragments, and consequently forming the basis of the final prediction results. While reviewing these predictions, occasional occurrences of motion fragments deviating from expected patterns within continuous motion sequences might be noticed. These fragments could be considered recognition errors or misclassified fragments, requiring postprocessing for correction and modification. In this study, if the predicted results of one fragment (situation 1) or two consecutive fragments (situation 2) differ from that of the preceding and subsequent fragments, and the predicted results of the adjacent fragments remain the same, the fragment is considered misclassified and adjusted to match the predicted results of the preceding fragment. The modification rule is shown in Figure 4. 

### 4.2. Volume Estimation

To evaluate the intake volume of a drinking activity, feature extraction and fluid intake regression are applied in volume estimation. In feature extraction, parameters that may affect the intake volume are surveyed. One of the most important parameters is sipping duration, which is highly related to the drinking volume. In addition, people rotate their wrist to tilt the container for sipping in the process of drinking water. The rotation of the wrist and the inclination of the container may be critical parameters for volume estimation. Features such as average inclination, maximum inclination, integral of inclination, number of samples with inclination over critical degrees, and numbers of samples with normalized inclination over critical percent of maximum inclination are extracted in three directions. Moreover, the initial fill level in a container may affect the motion while drinking water. A larger inclination angle is required while drinking with a container at a low fill level than that at a high fill level. The influence of fill level information on the performance of volume estimation should be analyzed. Table 3 shows 65 features for volume estimation extracted from signal of *sip* which is the process of water taking into mouth.

To estimate the fill level, support vector machine regression (SVR) using linear and Gaussian kernels are implemented. The applied features are the first 64 features shown in Table 3 except the fill level. These features are extracted from continuous signals from *pre-sip* to *post-sip* gestures. Fill levels in the container may influence the motion while using the container which is the period from pre-sip to post sip gestures. Therefore, signals of these gesture segments are used to extract features for fill level estimation to obtain the accurate fill levels.

In fluid intake regression, SVR models with linear and Gaussian kernels are used to estimate intake volume for the wrist-worn-based and smart-container-based approach. To analyze the effect of sip sizes on estimation, three sip-size-dependent SVR models are applied with three scales of sip sizes (small, medium, and large sip sizes). Four combinations of features and models are evaluated, including general SVR models using extracted features with and without fill levels and sip-size-dependent SVR models using features with and without fill levels.

### 4.3. Performacne Evaluation

The performance of the approaches is validated by leave-one-subject-out (LOSO) cross validation approach. To evaluate the performance of gesture recognition and volume estimation, several metrics are adopted. In gesture recognition, sensitivity, precision, F1-score, and accuracy are used for performance evaluation. These metrics can be calculated by Equations (1)–(4). The positive category presents the target gesture that needs to be recognized, while the negative category presents the other gestures. Then, true positive (*TP*) means the target gesture is correctly recognized as the target gesture. False positive (*FP*) means the other gesture is incorrectly recognized as the target gesture. True negative (*TN*) means the other gesture is correctly recognized as the other gestures. False negative (*FN*) means the target gesture is incorrectly recognized as the other gestures.

(1) $$Sensitivity=\frac{TP}{TP+FN}$$

(2) $$Precision=\frac{TP}{TP+FP}$$

(3) $$F1-score=\frac{2\times Sensitivity\times Precision}{Sensitivity+Precision}$$

(4) $$Accuracy=\frac{TP+TN}{TP+TN+FP+FN}$$

In volume estimation, the fill level and intake volume are estimated by regression models. Mean absolute percentage error (MAPE), mean absolute deviation (MAD) and coefficient of determination ($R^{2}$) are used to evaluate the performance of fill level estimation. MAPE, MAD, and root mean square error (RMSE) are used to evaluate the performance of volume estimation. The equations of MAPE, MAD, $R^{2}$, and RMSE are shown in Equations (5)–(8), where n is the number of intakes, ${\hat{a}}_{i}$ (is the estimated volume of *i*th intake, $a_{i}$ is the actual volume of *i*th intake and $\bar{a}$ is the average volume of intakes. MAPE calculates the percentage of absolute error between the actual intake volume and estimated intake volume. MAD calculates the absolute deviation between the actual and estimated intake volume. $R^{2}$ examines the reproducibility of the regression model. RMSE reveals the average difference of actual volume and estimated volume.

(5) $$MAPE=\frac{1}{n}\sum_{i=1}^{n}\left|\frac{{\hat{a}}_{i}-a_{i}}{a_{i}}\right|\times 100\%$$

(6) $$MAD=\frac{1}{n}\sum_{i=1}^{n}\left|{\hat{a}}_{i}-a_{i}\right|$$

(7) $$R^{2}=1-\frac{\sum_{i=1}^{n}{{(a}_{i}-{\hat{a}}_{i})}^{2}}{\sum_{i=1}^{n}{{(a}_{i}-\bar{a})}^{2}}$$

(8) $$RMSE=\sqrt{\frac{1}{n}\sum_{i=1}^{n}{\left({\hat{a}}_{i}-a_{i}\right)}^{2}}$$

## 5. Results

### 5.1. Gesture Recognition

**Figure 5:**
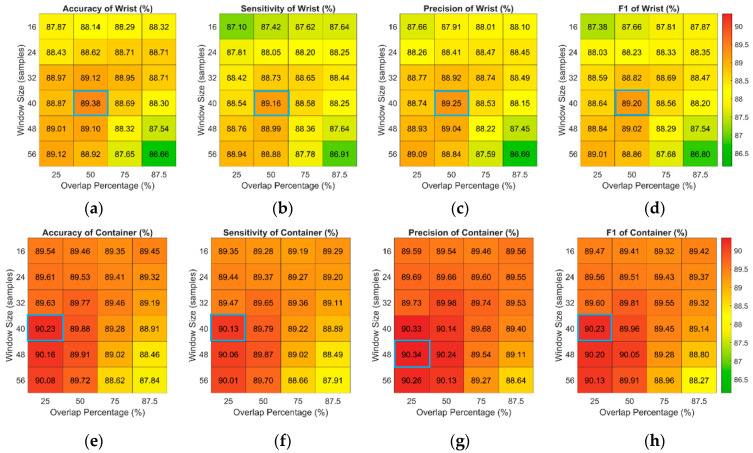
Recognition performance of (**a**) accuracy, (**b**) sensitivity, (**c**) precision, and (**d**) F1 score of pairwise combinations of window sizes and overlap percentages for the wrist-worn-based approach and (**e**) accuracy, (**f**) sensitivity, (**g**) precision, and (**h**) F1 score of pairwise combinations of window sizes and overlap percentages for the smart-container-based approach without postprocessing. The best performances of accuracy, sensitivity, and F1-score are marked with blue box.

**Figure 6:**
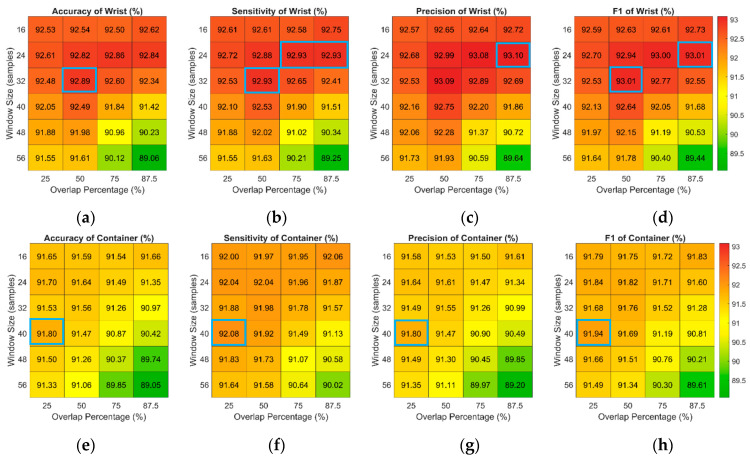
Recognition performance of (**a**) accuracy, (**b**) sensitivity, (**c**) precision, and (**d**) F1 score of pairwise combinations of window sizes and overlap percentages for the wrist-worn-based approach and (**e**) accuracy, (**f**) sensitivity, (**g**) precision, and (**h**) F1 score of pairwise combinations of window sizes and overlap percentages for the smart-container-based approach with postprocessing. The best performances of accuracy, sensitivity, and F1-score are marked with blue box.

The proposed fluid intake assessment approaches include gesture recognition and volume estimation. In gesture recognition, different combinations of window sizes and overlap percentages are explored. Figure 5 and Figure 6 demonstrate the results of gesture recognition using different combinations of window sizes and overlap percentages. Figure 5 shows the results of wrist-worn-based and smart-container-based gesture recognition without postprocessing. Without the postprocessing step, wrist-worn-based gesture recognition using a window size of 40 samples and an overlap percentage of 50% achieves the best performance of accuracy, sensitivity, precision, and F1-score, which are 89.38%, 89.16%, 89.25%, and 89.20%, respectively. For smart-container-based gesture recognition, using the window size of 40 samples and 25% overlapping can obtain the best accuracy, sensitivity, and F1-score, which are, respectively, 90.23%, 90.13%, and 90.23%. The precision of using the window size of 40 samples and 25% overlapping is 90.33% and is only slightly worse (0.01%) than that of using the window size of 48 samples and 25% overlapping. 

Figure 6 shows the results of gesture recognition with postprocessing. In wrist-worn-based gesture recognition, using the window size of 32 samples and the overlap percentage of 50% can achieve the best performance of accuracy, sensitivity, and F1-score, which is 92.89%, 92.93%, and 93.01%, respectively. Although the precision of this combination is 93.09%, the difference between the precision of that and the best precision of using 24 samples and 87.5% overlapping is only 0.01%. The best performance of smart-container-based gesture recognition is using the window size of 40 samples and the overlap percentage of 25%. This combination can obtain 91.80% accuracy, 92.08% sensitivity, 91.80% precision, and 91.94% F1-score.

### 5.2. Volume Estimation

Results of fill level estimation for the two approaches are shown in Table 4. The results demonstrate that the performance of SVR model using the linear kernel is better than that using the Gaussian kernel in fill level estimation. The SVR model using the linear kernel can achieve 29.89% MAPE and 56.30% MAD for the wrist-worn-based approach and 11.68% MAPE and 21.57% MAD for the smart-container-based approach. In addition, fill level estimation of the smart-container-based approach outperforms that of the wrist-worn-based approach. MAPE and MAD of the wrist-worn-based approach is about twice larger than that of the smart-container-based approach.

For volume estimation, different combinations of features and models are implemented to explore the important parameters. The results of these combinations are shown in Table 5, Table 6, Table 7 and Table 8. The best performance of general SVR models is 105.95% MAPE and 15.33 g MAD for models using features without fill levels and 105.64% MAPE and 15.6 g MAD for models using features with fill levels. The performance of general SVR models using the linear kernel is better than that using the Gaussian kernel. The difference between using the linear kernel and the Gaussian kernel is about 1 g in MAD and 25–40% in MAPE. For sip-size-dependent SVR models using features with and without fill levels, the performance of models using the Gaussian kernel is slightly better than that using the linear kernel except small-sip-size models. However, the difference between using the linear kernel and the Gaussian kernel in small-sip-size models is small. The difference of MAD is about 2 g and the difference of MAPE is within 3.5%.

Compared with general SVR models, sip-size-dependent SVR models achieve better performance. For the wrist-worn-based approach, the best performance of general SVR models is 105.64% MAPE, 15.60 g MAD, and 19.81 g RMSE, and the sip-size-dependent SVR models can reach the best performance of 15.52% MAPE, 7.40 g MAD, and 9.33 g RMSE for large-sip sizes, 22.21% MAPE, 5.43 g MAD, and 6.52 g RMSE for medium-sip sizes, and 94.18% MAPE, 5.03 g MADm and 6.05 g RMSE for small-sip sizes. For the smart-container-based approach, the best performance of the general SVR models is 106.24% MAPE, 15.74 g MAD, and 20.23 RMSE, and the performance of the sip-size-dependent SVR models can achieve 15.72% MAPE, 7.53 g MAD, and 9.44 g RMSE for large-sip sizes, 22.56% MAPE, 5.65 g MAD, and 6.76 g RMSE for medium-sip sizes, and 97.70% MAPE, 5.86 g MAD, and 6.79 g RMSE for small-sip sizes. The results demonstrate a great improvement in performance of sip-size-dependent SVR models. However, the performance of sip-size-dependent SVR models using features with fill levels is close to that using features without fill levels.

## 6. Discussion

To analyze the wrist-worn-based and smart-container-based approaches, this study uses inertial sensors worn on the wrist and attached to the container in the same experimental scenario. These two sensors collect the motion data of the wrist and the container while drinking water. Each approach utilizes a hierarchical algorithm to recognize drinking gestures and estimate intake volumes. Gesture recognition applies machine learning models to identify drinking gestures. Among the recognized gestures, sip gestures are highly correlated to drinking volumes. In volume estimation, regression models apply features extracted from sip gestures and fill level information to estimate intake volumes. To improve the estimation performance, three models of different sip sizes are employed. The results demonstrate that the wrist-worn-based approach and smart-container-based approach has similar performance in gesture recognition and volume estimation. However, different steps and factors may influence the performance.

Gesture recognition identifies grasp, pre-sip, sip, post-sip, and release for the wrist-worn-based approach and pre-sip, sip, and post-sip for the smart-container-based approach. The results demonstrate the recognition performance of the wrist-worn-based approach is worse than that of the smart-container-based approach without postprocessing, but the performance of the wrist-worn-based approach is better than that of the smart-container-based approach with postprocessing. The difference of accuracy, sensitivity, precision and F1-score between these two approaches is 0.85%, 0.97%, 1.08% and 1.02%, respectively. After postprocessing, the performance of wrist-worn-based recognition has large improvement while that of smart-container-based recognition shows a small increment. The accuracy, sensitivity, precision, and F1-score of wrist-worn-based recognition with post-processing are 1.09%, 0.85%, 1.29%, and 1.07% better than that of smart-container-based recognition. The improvement in accuracy, sensitivity, precision, and F1-score for the wrist-worn-based approach after postprocessing is 3.51%, 3.77%, 3.84%, and 3.81%. Postprocessing can effectively deal with the problem of misclassified fragments for the wrist-worn-based approach. The main reason for this is that the signal of the wrist-worn sensor has greater variability than the signal of the smart-container sensor. The wrist-worn sensor is more easily influenced by individual differences than the smart-container sensor, which is attached to the bottom of the container. Thus, more misclassified fragments may be involved in the continuous gesture fragments. Through the modification of postprocessing, these misclassified fragments can be correctly modified to improve the recognition performance for the wrist-worn-based approach.

To evaluate the influence of fill level information for estimation performance, different combinations of true data, recognized data, true fill levels and estimated fill levels are applied to the regression models. Table 9 shows the comparison results of these combinations. The results demonstrate that the recognized data affects the estimation performance greatly. In addition, the wrist-worn-based approach has a worse performance while using fill levels as features, but the smart-container-based approach achieves better performance while fill levels as features. For the wrist-worn-based approach with large variability, applying fill levels as features diminishes the estimation performance. That means the motion of the wrist is largely irrelevant to the fill level in the container. On the contrary, fill levels can improve the estimation performance for the smart-container-based approach. For the smart-container-based approach, it is not sensitive to the wrist motion but the container information. Therefore, the information of fill levels that is highly correlated to the container is more important.

Sip-size-dependent SVR models are implemented to analyze the importance of sip size information. The results of the sip-size-dependent SVR models demonstrate that the performance can be greatly improved compared with general SVR models. Although the MAPE of models for small sip sizes is over 90%, the improvement of MAD and RMSE for small sip sizes can reach over 50%. The large value of MAPE is because of the small actual intake volume. Because a smaller actual volume leads to a larger value of MAPE for small sip sizes, MAD and RMSE are more important metrics. The mean and standard deviation of actual volume for large, medium, and small sip sizes are 53.82 g ± 8.58 g, 27.23 g ± 6.20 g, and 10.71 g ± 5.56 g. The MAD of these models are close to the standard deviation of actual intake volume.

The wrist-worn-based approach and smart-container-based approach have similar estimation performances. However, information of fill levels and sip sizes have different effects on the wrist-worn-based approach and smart-container-based approach. For the wrist-worn-based approach, information of sip sizes is more important. As shown in Table 9, using the recognized data reduces the estimation performance, but using fill levels as features causes a great decline of the estimation performance. This decline can be improved by applying sip-size-dependent SVR models. For the smart-container-based approach, fill levels and sip sizes are both important information. Although the recognition performance affects the estimation performance, using actual fill levels can improve the performance. Moreover, sip-size-dependent SVR models can enhance the estimation performance. The estimation performance of the sip-size-dependent SVR models is insusceptible to fill levels.

## 7. Conclusions

This study aims to analyze wrist-worn based and smart-container-based fluid intake assessment approaches using inertial sensors in the same experimental scenario. A hierarchical algorithm is applied to recognize drinking gestures and estimate intake volumes. The accuracy of gesture recognition with postprocessing is 92.89% and 91.8% for the wrist-worn-based approach and smart-container-based approach, respectively. For volume estimation, sip-size-dependent SVR models can achieve better performance than general SVR models for both wrist-worn-based and smart-container-based approaches. The improvement of MAPE, MAD, and RMSE can reach over 50% except MAPE for small sip sizes. Using fill levels as features has no effect on the performance for recognized data while the fill level information enhances the estimation performance of the smart-container-based approach if using true data. However, the estimation performance of the sip-size-dependent SVR models is insusceptible to fill levels. The results demonstrate that the sip size information and gesture recognition performance are important for fluid intake assessment approaches.

## Figures and Tables

**Table 1:** Literature of wrist-worn-based and smart-container-based system fluid intake assessment using various algorithms and sensors.

System Type	Article (Year)	Algorithm	Sensor	Placement/Position	Results
Wrist-worn-based	Hamatani et al. (2018) [2]	Drinking activity recognition, gesture spotting and intake volume estimation	Smartwatch	Dominant hand	Drinking activity recognition: 83.6% precision and 87.3% sensitivity Sip gesture spotting: 90.7% precision and 96.3% sensitivity Volume estimation: 29.1% MAPE ^1^ and 15.1% MPE ^1^
	Chun et al. (2019) [23]	Drinking activity detection	Inertial sensor	Left and right wrists	Precision: 90.3% Sensitivity: 91.0%
	Gomes and Sousa (2019) [16]	Pre-drinking (hand-to-mouth) movement recognition	Inertial sensor	Dominant wrist	F-score: 97% in an offline validation and 85% in a real-time free-living validation
	Huang et al. (2020) [22]	Drinking activity recognition, gesture spotting and intake volume estimation	Inertial sensor	Right wrist	Drinking activity recognition: 94.42% accuracy Gesture spotting: 90.17% sensitivity Volume estimation: 40.11% MAPE ^1^
	Ortega Anderez et al. (2021) [15]	Eat and drink intake recognition	Accelerometer	Dominant hand	Eat intake recognition: 96.29% accuracy, 87.43% precision, and 95.81% sensitivity Drink intake recognition: 98.73% accuracy, 92.98% precision, and 89.83% sensitivity
Smart-container-based	Griffith et al. (2019) [18]	Micro-event partitioning and volume estimation	Inertial sensor	Bottle	Volume estimation: 52.39% MAPE ^1^
	Griffith et al. (2019) [24]	Container type and fill level classification	Inertial sensor	Bottle, glass and mug	Container type classification: 97.3% and 77.3% accuracy at the two fill levels considered Fill level classification: 100% accuracy in glass and mug container, and 98.0% accuracy in bottle
	Liu et al. (2020) [25]	Drinking event detection and episode identification	Inertial sensor	3D-printed smart cup	Event identification: 89.92% and 85.88% F-measure in event-defined and frame-defined evaluation approaches
	Cohen et al. (2022) [19]	Intake volume estimation	Capacitive or pressure sensors	Bottle	commercial bottle: H2Opal: −2.10% MPE ^1^ HidrateSpark steel: −16.11% MPE ^1^ HidrateSpark 3: −14.9% MPE ^1^ Thermos Smart Lid: 14.64% MPE ^1^

^1^ MAPE: mean absolute percentage error; MPE: mean percentage error.

**Table 2:** The list of features extracted for gesture recognition. Eight statistical features are applied, including mean, standard deviation, variance, maximum, minimum, range, skewness, and kurtosis to extract from tri-axial acceleration, angular velocity, angular acceleration, and inclination.

Feature	Description
$f_{1}$–$f_{12}$	Mean of tri-axial acceleration, angular velocity, angular acceleration, and inclination
$f_{13}$–$f_{24}$	Standard deviation of tri-axial acceleration, angular velocity, angular acceleration, and inclination
$f_{25}$–$f_{36}$	Variance of tri-axial acceleration, angular velocity, angular acceleration, and inclination
$f_{37}$–$f_{48}$	Maximum of tri-axial acceleration, angular velocity, angular acceleration, and inclination
$f_{49}$–$f_{60}$	Minimum of tri-axial acceleration, angular velocity, angular acceleration, and inclination
$f_{61}$–$f_{72}$	Range of tri-axial acceleration, angular velocity, angular acceleration, and inclination
$f_{73}$–$f_{84}$	Skewness of tri-axial acceleration, angular velocity, angular acceleration, and inclination
$f_{58}$–$f_{96}$	Kurtosis of tri-axial acceleration, angular velocity, angular acceleration, and inclination

**Table 3:** The list of features extracted for volume estimation. Seven feature types are applied, including duration, average inclination, maximum inclination, integral inclination, number of samples with inclination over nine different degrees, number of samples with normalized inclination over nine different percentages of maximum inclination, and fill level.

Feature	Description
$f_{1}$	Duration
$f_{2}$–$f_{4}$	Average inclination in 3 directions
$f_{5}$–$f_{7}$	Maximum inclination in 3 directions
$f_{8}$–$f_{10}$	Integral of inclination in 3 directions
$f_{11}$–$f_{37}$	Number of samples with inclination over 𝑘 degree in 3 directions 𝑘 ∈ {10°, 20°, 30°, 40°, 50°, 60°, 70°, 80°, 90°}
$f_{38}$–$f_{64}$	Number of samples with normalized inclination over p percent of maximum inclination in 3 directions p ∈ {10%, 20%, 30%, 40%, 50%, 60%, 70%, 80%, 90%}
$f_{65}$	Fill level

**Table 4:** The performance of $R^{2}$, MAD, and MAPE of wrist-worn-based and smart-container-based fill level estimation using SVR models with linear and Gaussian kernels on recognized data.

	Wrist-Worn-Based Approach		Smart-Container-Based Approach	
	SVR-Linear	SVR-Gaussian	SVR-Linear	SVR-Gaussian
$R^{2}$	0.49	0.01	**0.84**	0.17
MAD (g)	56.30	84.89	**21.57**	76.81
MAPE (%)	29.89	45.86	**11.68**	42.42

Note: bold fonts indicate the best performance.

**Table 5:** The performance of RMSE, MAD, and MAPE of wrist-worn-based volume estimation by General SVR models using different kernels and features.

Model	General SVR Model—Linear Kernel		General SVR Model—Gaussian Kernel	
	Without Fill Level	With Fill Level	Without Fill Level	With Fill Level
RMSE (g)	19.54	19.81	19.45	**19.33**
MAD (g)	**15.33**	15.60	16.56	16.54
MAPE (%)	105.95	**105.64**	146.07	146.63

Note: bold fonts indicate the best performance.

**Table 6:** The performance of RMSE, MAD, and MAPE of wrist-worn-based volume estimation by sip-size-dependent SVR models using different kernels and features.

Model	Sip-Size-Dependent SVR Model—Linear Kernel						Sip-Size-Dependent SVR Model—Gaussian Kernel					
	Without Fill Level			With Fill Level			Without Fill Level			With Fill Level		
	Large	Medium	Small	Large	Medium	Small	Large	Medium	Small	Large	Medium	Small
RMSE (g)	10.08	7.47	7.27	10.68	7.93	6.89	9.33	6.52	6.09	9.34	6.53	**6.05**
MAD (g)	8.02	6.19	5.77	8.48	6.56	5.31	7.40	5.43	5.05	7.40	5.43	**5.03**
MAPE (%)	15.88	23.76	99.99	16.69	25.13	93.37	**15.52**	22.21	94.45	15.54	22.24	94.18

Note: bold fonts indicate the best performance.

**Table 7:** The performance of RMSE, MAD, and MAPE of smart-container-based volume estimation by general SVR models using different kernels and features.

Model	General SVR Model—Linear Kernel		General SVR Model—Gaussian Kernel	
	Without Fill Level	With Fill Level	Without Fill Level	With Fill Level
RMSE (g)	20.12	20.23	19.80	**19.67**
MAD (g)	15.75	**15.74**	16.70	16.59
MAPE (%)	106.93	**106.24**	131.10	133.16

Note: bold fonts indicate the best performance.

**Table 8:** The performance of RMSE, MAD and MAPE of smart-container-based volume estimation by sip-size-dependent SVR models using different kernels and features.

Model	Sip-Size-Dependent SVR Model—Linear Kernel						Sip-Size-Dependent SVR Model—Gaussian Kernel					
	Without Fill Level			With Fill Level			Without Fill Level			With Fill Level		
	Large	Medium	Small	Large	Medium	Small	Large	Medium	Small	Large	Medium	Small
RMSE (g)	11.55	7.83	6.79	11.73	8.08	6.98	9.57	6.83	6.40	9.44	6.76	**6.28**
MAD (g)	9.17	6.38	5.86	9.32	6.58	6.08	7.63	5.73	5.28	7.53	5.65	**5.22**
MAPE (%)	18.78	24.21	97.70	19.06	25.08	103.25	15.92	22.78	99.08	**15.72**	22.56	98.15

Note: bold fonts indicate the best performance.

**Table 9:** The estimation performance of RMSE, MAD, and MAPE by wrist-worn-based and smart-container-based approaches using different combinations of true data, recognized data, true fill levels, and estimated fill levels.

Approach	Wrist-Worn-Based Approach				Smart-Container-Based Approach			
Data	True	Recognized	True	Recognized	True	Recognized	True	Recognized
Fill Level	--	--	True	Estimated	--	--	True	Estimated
RMSE (g)	18.80	19.54	18.79	19.81	20.00	20.12	**16.63**	20.23
MAD (g)	15.06	15.33	15.23	15.60	15.48	15.75	**11.29**	15.74
MAPE (%)	96.86	105.95	102.77	105.64	93.78	106.93	**71.18**	106.24

Note: bold fonts indicate the best performance.

## Data Availability

The data are not publicly available due to privacy or ethical restrictions.
